# Non-ambulatory pigs in two Brazilian growing-finishing farms: a clinic, etiological and pathological perspective on 76 cases

**DOI:** 10.1186/s40813-022-00279-6

**Published:** 2022-08-10

**Authors:** Manoela Marchezan Piva, Claiton I. Schwertz, Luan Cleber Henker, Ronaldo Michel Bianchi, Regina Tose Kemper, Bruno Albuquerque de Almeida, Ricardo Yuiti Nagae, Taís Regina Michaelsen, Saulo Petinatti Pavarini

**Affiliations:** 1 Departament of Veterinary Pathology, Faculty of Veterinary Medicine, Federal University of Rio Grande Do Sul - UFRGS, 9090 Av. Bento Gonçalves, Porto Alegre, Rio Grande Do Sul 91540-000 Brazil; 2 Animal Health Laboratory, Seara Alimentos LTDA, 155 Av. Paludo, Industrial, Seara, Santa Catarina 89770-000 Brazil

**Keywords:** Swine pathology, Locomotor disorders, Neurological diseases, Tail biting lesion

## Abstract

**Background:**

Non-ambulatory pigs, colloquially known as downers or downed pigs, are animals presented with limited to no mobility, usually as a result of pre-existing neurologic or musculoskeletal conditions. Impaired ambulation is a major cause of euthanasia in pigs, leading to economic losses and animal welfare concerns. Additionally, reaching the underlying diagnosis of impaired ambulation in pigs is commonly a challenging task for swine practitioners. The aim of this necropsy-based study was to report the clinical, etiological, and pathological findings of 76 non-ambulatory grower-finisher pigs, and to correlate tail-biting lesions with the causes of death/reason for euthanasia in non-ambulatory pigs. Necropsies of downed pigs were performed during on-site visits to two pig farms in southern Brazil.

**Results:**

The diagnosis of the conditions was based on the clinical, macroscopic, histopathological, bacteriological, immunohistochemical, and molecular findings. The diseases diagnosed in non-ambulatory pigs in this study were suppurative arthritis (29/76), suppurative spondylitis (10/76), PVC-2 associated diseases (8/76), bone fracture (7/76), non-suppurative meningoencephalomyelitis (4/76), suppurative meningoencephalitis (6/76), fibrocartilaginous thromboembolism (3/76), epiphysiolysis (3/76), ascending bacterial myelitis (3/76), and other conditions (3/76). The frequency of suppurative arthritis, suppurative spondylitis, and ascending bacterial myelitis/meningitis was higher in pigs with tail biting lesions than controls (*P* < 0.001).

**Conclusions:**

Non-ambulatory pigs were observed during the entire rearing period, however, the occurrence of non-ambulatory pigs increased in animals aged ≥ 150 days. Infectious diseases were the most common cause of downed pigs, mainly associated with chronic bacterial infections. Tail biting lesions were an important predisposing factor to suppurative arthritis, suppurative spondylitis, and ascending bacterial myelitis/meningitis.

## Background

Pigs presenting significant locomotion deficits, including difficulty to stand up and inability to walk are known as non-ambulatory pigs [1]. Non-ambulatory pigs may be colloquially referred to as "downers" or "downed" pigs and represent a recurring problem in commercial pig farms worldwide [2]. Non-ambulatory pigs may be observed as a result of a plethora of diseases affecting primarily the Central Nervous System (CNS) and the musculoskeletal system, including infectious, nutritional, traumatic, toxic, and degenerative conditions [3].

Non-ambulatory pigs frequently die or are euthanized in the farms due to poor prognosis. Additionally, partial or total carcass condemnation due to disseminated lesions are common outcomes at the instances where these pigs arrive at the slaughterhouse [1]. These lesions are reported to be largely associated with tail lesions in swine [4]. Furthermore, non-ambulatory pigs are estimated to cost 46 million dollars annually to the U.S. swine industry [1]. This problem had an estimated cost of $54.91 per non-ambulatory pig in the US between 2012 and 2015 and affected 0.63% of pigs slaughtered between those years [5].

Besides economic losses, non-ambulatory pigs represent a major concern regarding animal welfare standards in pig farms. Impaired ambulation may lead to insufficient food and water consumption and may favor injuries occurring as a result of negative interactions with other pigs. Additionally, several diseases leading to impaired ambulation are painful and impact negatively normal pig behavior [1].

This type of clinical manifestation bas been frequently reported in sows, comprising the cause of death/main reason for euthanasia of 13–23.9% of animals in this category in previous studies [2, 6, 7]. Diseases leading to impaired ambulation and lameness are also common in rearing pigs, however, comprehensive studies documenting the etiologic and pathologic findings observed in these cases are limited.

Investigating the underlying causes for impaired ambulation may be challenging for swine practitioners due to the great variety of possible causes, the manifestation of nonspecific clinical signs and the need for a systematic and detailed assessment of the CNS and the musculoskeletal system in these cases. Thus, it is plausible to assume that the underlying cause for impaired ambulation in pigs remains undetermined in many, if not most cases occurring in the farms. In this scenario, the necropsy examination represents an important tool to investigate and elucidate the main causes of impaired ambulation in pigs, yielding crucial information to measure and monitor continuous improvements for animal welfare in the swine industry and mitigate economic losses.

In a previous work, we assessed the causes of mortality in the growing-finishing phase in two pig farms in Southern Brazil [8]. In this necropsy-based study, 610 pigs were evaluated, 76 (12.5%) of which were non-ambulatory pigs. In the current study, the aim was to describe in greater depth and with visual resources the clinical, etiological, and pathological aspects of this subset of cases. In addition, analysis to correlate tail-biting lesions with the causes of death/reason for euthanasia in non-ambulatory pigs were conducted.

## Results

**Fig. 1:**
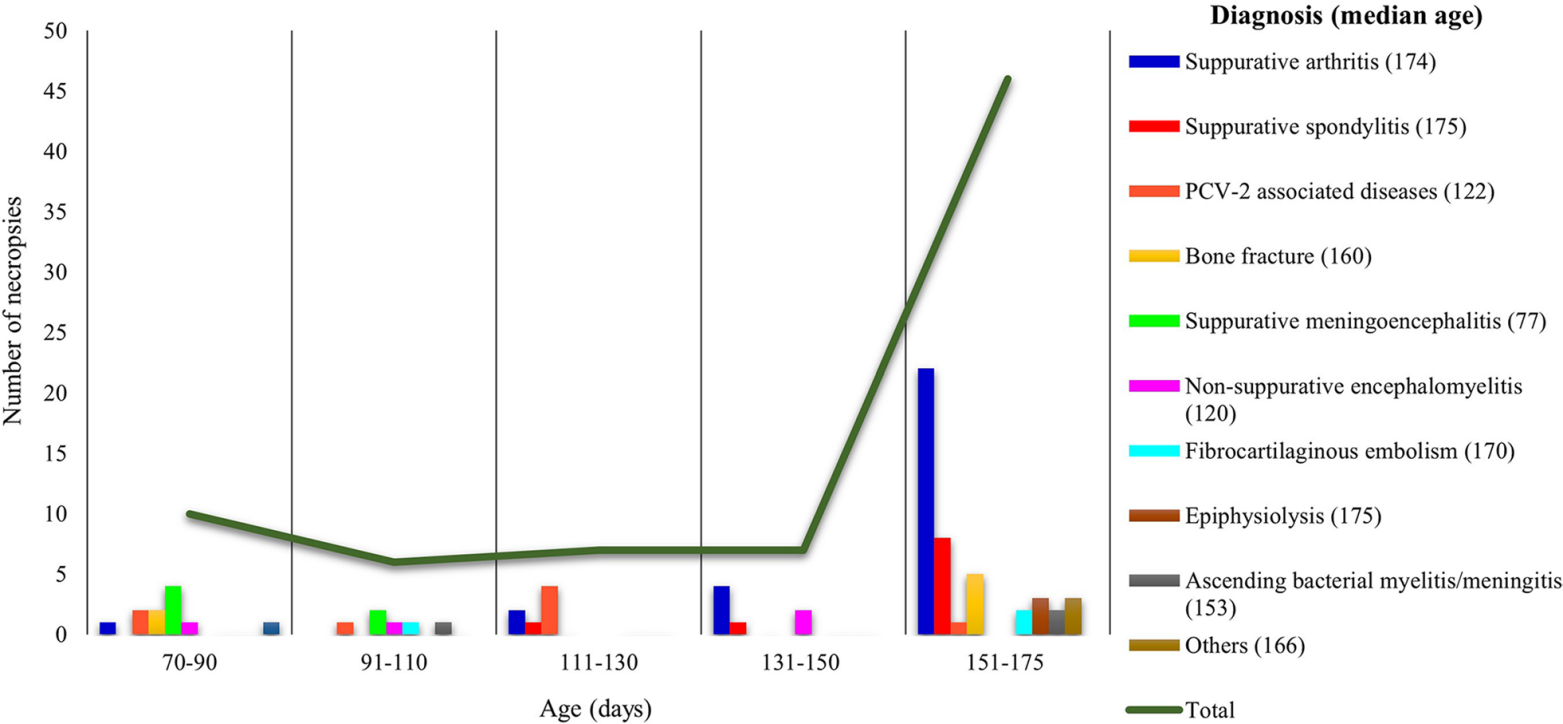
Distribution of 76 non-ambulatory growing-finishing pigs submitted for necropsy according to age group categories and *post-mortem* diagnosis

The underlying cause for impaired ambulation was infectious in 61/76 pigs (80.2%) and non-infectious in 15/76 (19.8%). Fourteen different pathological entities were diagnosed based on the necropsy findings. Information on the frequency of diagnosis and distribution according to sex, Body Condition Score (BCS) and frequency of tail-biting lesions are shown in Table 1. The distribution of diseases is depicted according to age group categories in Fig. 1, and it is possible to observe a greater involvement of animals at the end of the finishing phase (≥ 150 days).

**Table 1:** Postmortem diagnoses of 76 non-ambulatory pigs in the growing-finishing phase

Diagnosis	Incidence				Tail-biting lesion		EBC	N/E
	%	N	Barrows	Females	%	n		
Suppurative arthritis	38.2	29/76	24	5	44.82	13/29	2	7/22
Suppurative spondylitis	13.2	10/76	7	3	60	6/10	2	2/8
PCV-2 associated diseases	10.5	8/76	1	7	0	0/8	3	2/6
Bone fracture	9.2	7/76	3	4	14.2	1/7	3	0/7
Suppurative meningoencephalitis	8	6/76	3	3	16.6	1/6	3	4/2
Non-suppurative encephalomyelitis	5.3	4/76	2	2	0	0/4	3	1/3
Fibrocartilaginous embolism	3.9	3/76	1	2	0	0/3	4	2/1
Epiphysiolisis	3.9	3/76	2	1	0	0/3	3,5	1/2
Ascending bacterial myelitis/meningitis	3.9	3/76	2	1	100	3/3	2,5	1/2
Others	3.9	3/76	2	1	0	0/3	3	2/1
Total	100	76	47	29	31.8	24/76		

n: number; %: percentage; BCS: Body Condition Score; N/E: natural death / euthanasia

The proportion of pigs with tail-biting associated diseases (suppurative arthritis, suppurative spondylitis and ascending bacterial myelitis/meningitis) differed (Pearson's Chi-square test *P* value < 0.001) between pigs with (67.74%) and without tail-biting lesions (0.04%). Pigs with tail-biting lesions were 56.7 times (95% CI 12.5 to 256.3) more likely to have suppurative arthritis, suppurative spondylitis, or ascending bacterial myelitis/meningitis than controls (Logistic regression *P* value < 0.01).

Despite the high frequency of barrows among affected pigs, the proportion of tail-biting lesions did not differ (Fisher Exact *P* value = 0.720) between barrows (51.51%) and females (65.50%). Information for individual entities diagnosed during this study is described in the following subsections.

### Suppurative arthritis and spondylitis

**Fig. 2:**
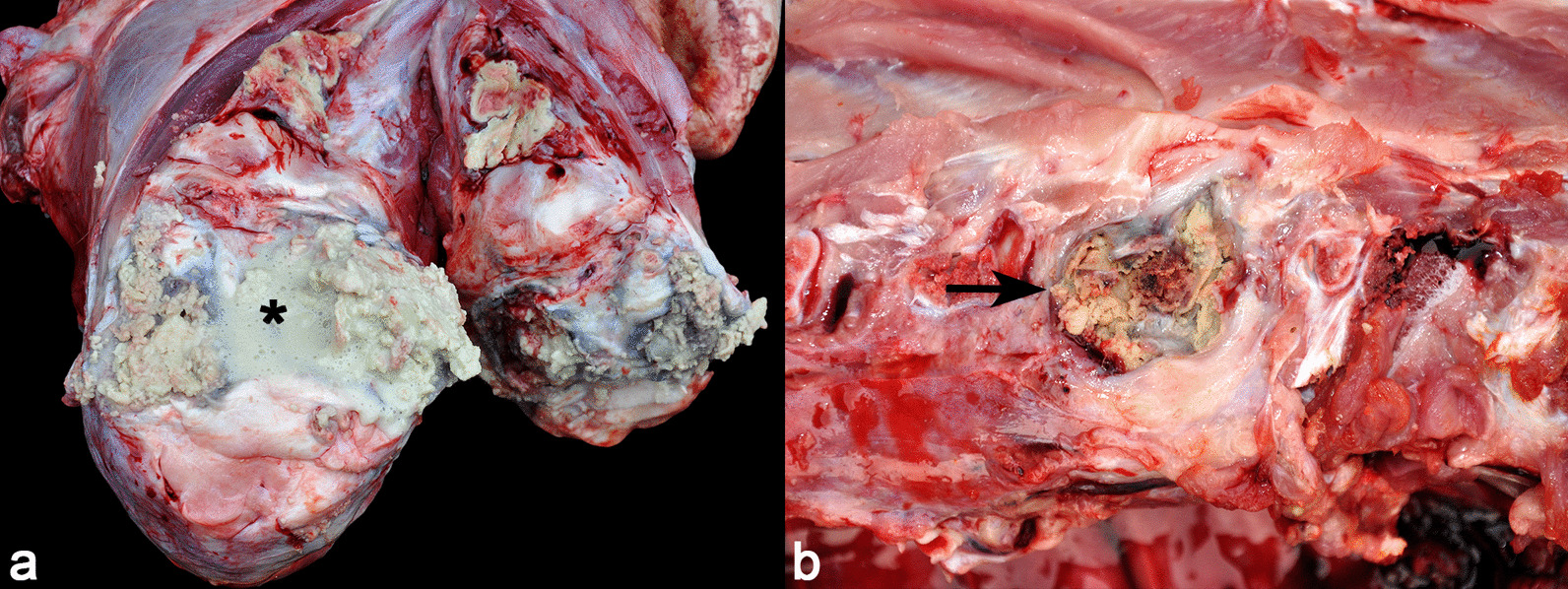
Suppurative arthritis and spondylitis in pigs. **a** Femoro-tibio-patellar joint cavity filled with oozing purulent material (suppurative arthritis) (asterisk). **b** Vertebral body with a focal area containing purulent yellowish material (pus) surrounded by a fibrous capsule (suppurative spondylitis) (arrow)

Pigs diagnosed with suppurative arthritis presented non-weight-bearing lameness, enlarged joints, and reluctance or impossibility to stand up and walk. The clinical course was predominantly chronic. Grossly, affected joints were enlarged and filled with purulent material. In many cases, the joint was surrounded by fibrous connective tissue, with the formation of periarticular abscesses (Fig. 2a). The most commonly affected joint in this condition was the femoral-tibia-patellar (16/29), followed by the tarsal joint (8/29), humerus-radio-ulnar (6/29), coxofemoral (5/29), costochondral (4/29), scapula-humerus (3/29), metacarpal-phalanx, interphalangeal and tibiotarsal (2/29 each), and carpal joint (1/29). The lesion was restricted to a single joint in 11/29 pigs, and the involvement of two or more joints was seen in 18/29.

Microscopically, there was marked necrosis of the synovial membrane, accompanied by fibrinosuppurative exudate, aggregates of bacterial miriadis, and proliferation of fibrous connective tissue. Bacterial culture was obtained in thirteen cases (13/29), with identification of *Pasteurella multocida* type D (4/29), *Trueperella pyogenes* (4/29), *Staphylococcus* spp. (2/29), and *Streptococcus suis* (2/29).

Cases of spondylitis affected pigs in the final period of the finishing phase. The main clinical signs observed were paresis/paralysis of the pelvic limbs and inability to stand up. Lesions were observed in the lumbosacral (5/10) and thoracic (5/10) segments of the vertebral column. Concomitant suppurative lesions found in pigs with spondylitis included arthritis (5/10) and embolic pneumonia (3/10).

Gross lesions in cases of spondylitis consisted of a single focally extensive area of enlargement in the vertebral column, surrounded by a thick white and firm capsule, containing pus that involved and infiltrated the affected vertebrae (Fig. 2b). Microscopically, these lesions were characterized by osteonecrosis, accompanied by fibrinosuppurative inflammation, coccoid bacterial aggregates, and proliferation of fibrovascular tissue in the surrounding area. This diagnostic group was characterized by chronic clinical course. Bacterial culture was obtained in two of the ten samples with isolation of *Staphylococcus* spp. and *Pasteurella multocida* (one case each).

### Porcine Circovirus type 2 (PCV-2) associated disease

PCV-2 associated disease was diagnosed as the underlying cause for impaired ambulation in eight pigs (8/76), with the presence of two classic clinical syndromes, Porcine Dermatitis and Nephropathy Syndrome (PDNS) (6/8), and Post-weaning Multisystemic Wasting Syndrome (PMWS) (2/8). Affected pigs had subacute to chronic clinical signs, with prolonged lateral recumbency (4/8), muscle tremors (2/8), and ataxia (2/8).

**Fig. 3:**
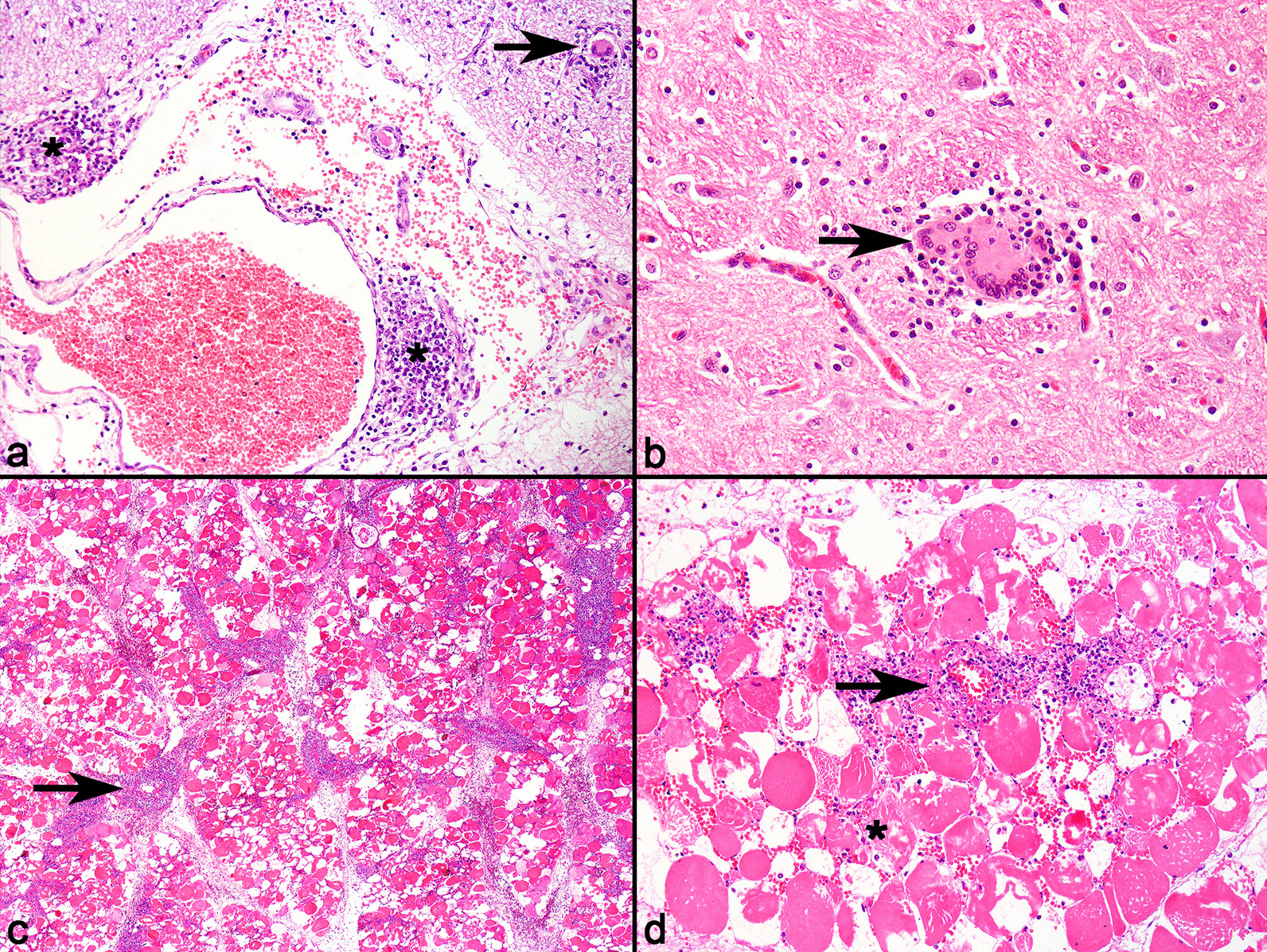
Lymphohistiocytic meningoencephalitis and necrotic myositis due to PCV-2 associated disease in pigs. **a** Telencephalic cortex. Moderate perivascular inflammatory infiltrate of lymphocytes and plasma cells segmentally expanding the leptomeninges, sometimes forming small perivascular aggregates (asterisk), accompanied by mild hemorrhage. Areas with accumulations of rare lymphocytes and multinucleated giant cells are observed in the adjacent neuropil (arrow). HE. × 40. **b** Area with mild infiltrate of lymphocytes, multinucleated giant cells, and glial cells (arrow), adjacent to a blood vessel. HE. × 200. **c** Skeletal muscle with marked diffuse necrosis, with intense vasculitis (arrow), and multifocal interstitial inflammatory infiltrate. HE. × 40. **d** Skeletal muscle with marked diffuse biphasic necrosis (asterisk), with intense vasculitis (arrow), and multifocal inflammatory infiltrate of neutrophils and lymphocytes, accompanied by interstitial hemorrhage. HE. × 100

Affected pigs had different combinations of lesions that are typical of the syndromes associated with PCV-2 infection, including granulomatous lymphadenitis, interstitial nephritis, and pneumonia. Additionally, in pigs with PCV-2 associated disease that were recumbent, inflammatory lesions were observed in the central nervous system (CNS) (8/8) and skeletal musculature (5/8). Neurologic lesions were characterized by non-suppurative meningoencephalomyelitis (4/8), and vasculitis involving mainly blood vessels of the neuropil and leptomeninges (8/8). The CNS inflammatory component in these cases was comprised by macrophages, lymphocytes (Fig. 3a), fewer multinucleated giant cells (3/8), eosinophils (1/8), and multifocal areas of gliosis (2/8) (Fig. 3b). The inflammatory infiltrate often formed small nodular aggregates surrounding blood vessels (Fig. 3a).

Muscle changes in cases of PCV-2 associated disease were grossly characterized by pale areas interspersed with petechiae predominantly in muscle fascias (3/5), mainly in the pelvic (4/5) and thoracic limbs (3/5). Histologically, affected muscles had biphasic muscle necrosis of varying degrees, with intense vasculitis, hemorrhage, and inflammatory infiltrate of neutrophils, lymphocytes, macrophages, and multinucleated giant cells (Fig. 3c and d). Six samples sent to PCR for PCV-2 were positive (6/6). IHC for PCV-2 carried out using sections of CNS of affected pigs detected positive immunolabeling in only one case (positive immunolabeling in lymphocytes and endothelial cells) (1/8). IHC for PCV-2 conducted using sections of the muscle of affected pigs was negative in all cases (5/5). However, all cases of PCV-2 associated disease had positive results for IHC for PCV-2 in sections of lymph nodes (8/8).

### Bone fractures

Pigs with bone fractures had clinical signs of acute or peracute onset, mainly characterized by paralysis of the pelvic limbs and inability to stand up. Pigs in this study had vertebral fractures (6/7), distributed in the lumbosacral segment (L5, L6, and sacrum) (3/6), followed by the thoracic segment (T7–T8 and T10) (2/6), and the lumbar segment (L2–L3) (1/6). In one case the fracture was observed in the humerus (1/7). Macroscopically, vertebral fractures were associated with marked subdural hemorrhage. Bone repair changes and hemorrhage were seen on histology. Additionally, the spinal cord had microscopic findings consistent with Wallerian degeneration in two cases, compatible with spinal cord compression secondary to bone fracture and subdural hemorrhage. Only subdural hemorrhage was observed in the remaining cases.

### Suppurative meningoencephalitis

**Fig. 4:**
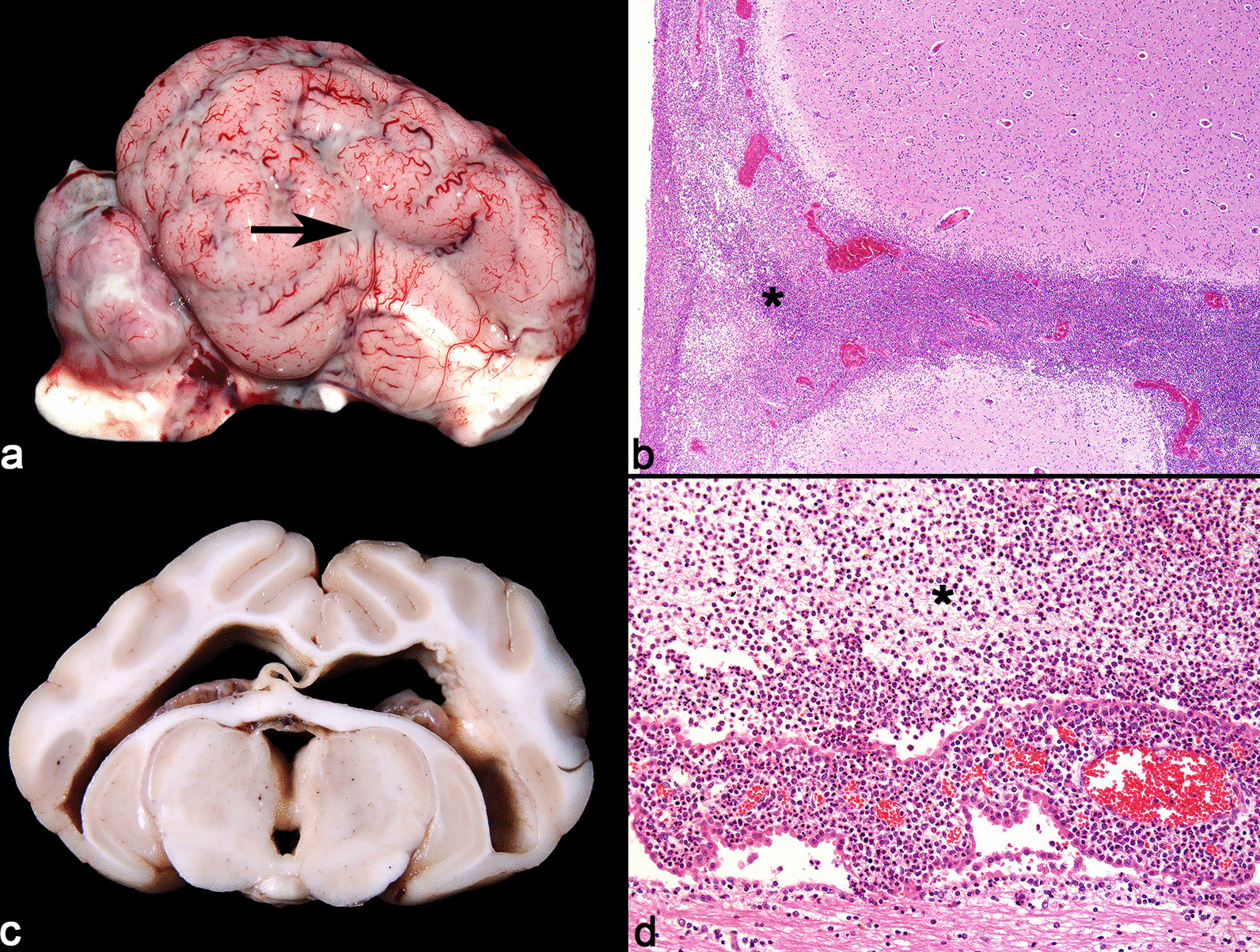
Suppurative meningoencephalitis in pigs. **a** Moderate, diffuse deposition of white material on the surface of the leptomeninges (arrow), accompanied by engorgement of leptomeningeal vessels and moderate hyperemia of the telencephalic cortex. **b** Telencephalic cortex with markedly thickened leptomeninges, with intense infiltrate comprised predominantly by neutrophils, fibrin deposition and marked diffuse congestion (asterisk). HE. × 40. **c** Cut surface of the brain, with intense dilation of lateral ventricles, third ventricle and midbrain aqueduct (hydrocephalus). On the ependymal surface, there is deposition of an opaque and irregular whitish material. d. Ventricle, ependyma and choroid plexus obscured and effaced by marked neutrophilic inflammatory infiltrate and fibrin deposition (suppurative ependymitis and plexochoroiditis) (asterisk). HE. × 100

Six pigs with suppurative meningoencephalitis of probable bacterial origin were assessed. The main clinical signs were motor incoordination, lateral recumbency, and convulsions, predominantly with acute evolution. Gross findings included leptomeningeal hyperemia and fibrin deposition in the brain (2/6), and the remaining organs were unremarkable (Fig. 4a). The main microscopic finding was fibrinosuppurative leptomeningitis with occasional coccoid bacterial aggregates (Fig. 4b). Bacterial isolation from CNS sample was successful in only one case, with the identification of *Streptococcus suis*.

Valvular endocarditis caused by *Streptococcus suis* was observed concomitantly with PCV-2 associated systemic disease in two cases of pigs diagnosticated with suppurative meningoencephalitis. One of these cases also presented a marked non-communicating hydrocephalus as a result of a bacterial fibrinosuppurative meningo-chorio-ependymitis (Fig. 4c and d).

### Non-suppurative encephalomyelitis

**Fig. 5:**
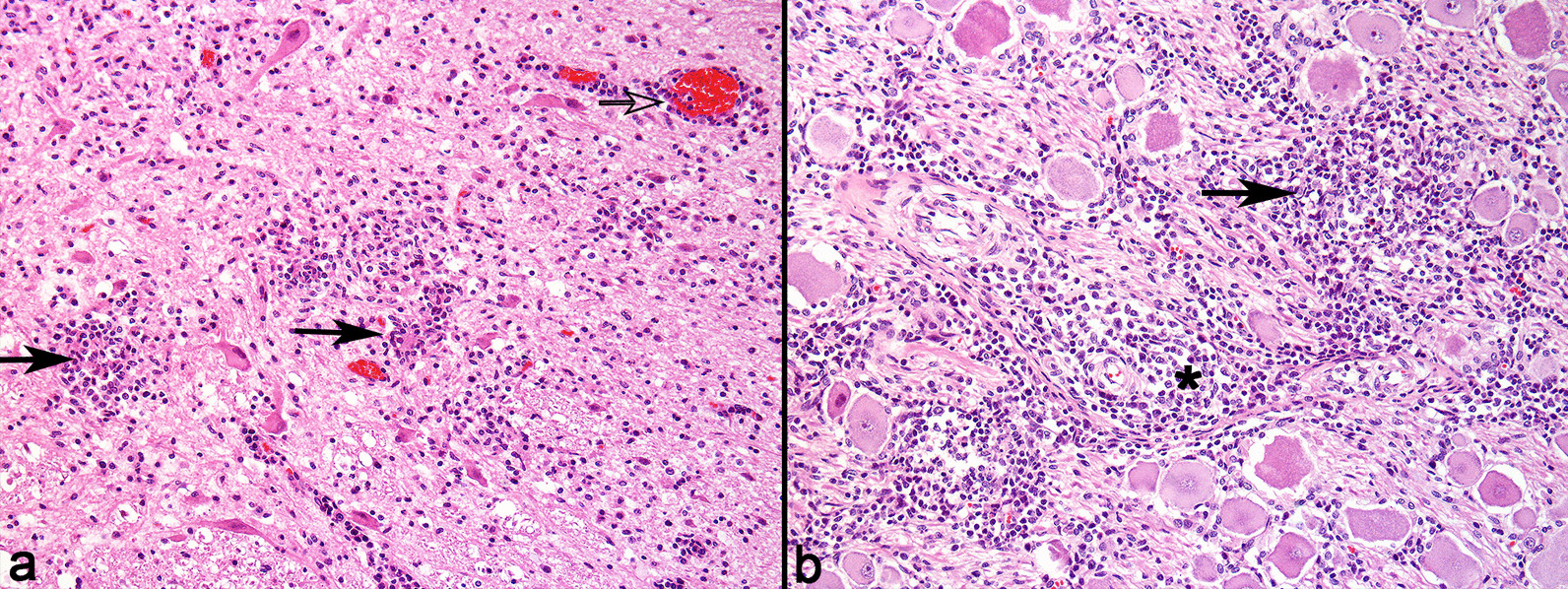
Non-suppurative encephalomyelitis in pigs. **a** Spinal cord, grey matter with moderate perivascular infiltrate of lymphocytes (transparent arrow), plasma cells and multifocal necrotic neurons accompanied by areas of gliosis and neuronophagia (arrows). HE. × 40. **b** Paravertebral ganglia with lymphoplasmacytic inflammatory infiltrate (ganglioneuritis) (asterisk), multifocal necrotic neurons and neuronophagia (arrow). HE. × 40

Non-suppurative encephalomyelitis (NEM), with distinct lesions from cases of PCV2-associated neurologic disease was diagnosed in four cases. Pigs in this category had clinical signs of acute onset, and subacute to chronic clinical course, with manifestation of lateral decubitus, paralysis or tetraparesis, ataxia and convulsions. Gross lesions were absent. Microscopic findings included moderate lymphoplasmacytic perivascular infiltrate in several sections of the brain and meninges (4/4), in all segments of spinal cord (3/4) and choroid plexus (1/4), accompanied by multifocal gliosis (3/4) and neuronophagia (2/4) (Fig. 5a). Lymphoplasmacytic ganglioneuritis with Wallerian degeneration and occasional neuronophagia were also observed in cases with spinal cord lesions (Fig. 5b). In the brain, the lesions were more prominent in the brainstem, while in the spinal cord, they were more prominent in the gray matter. Swabs of the meninges and/or samples of cerebrospinal fluid were collected in all four cases, and no significant bacterial growth was cultured.

### Fibrocartilaginous embolism

**Fig. 6:**
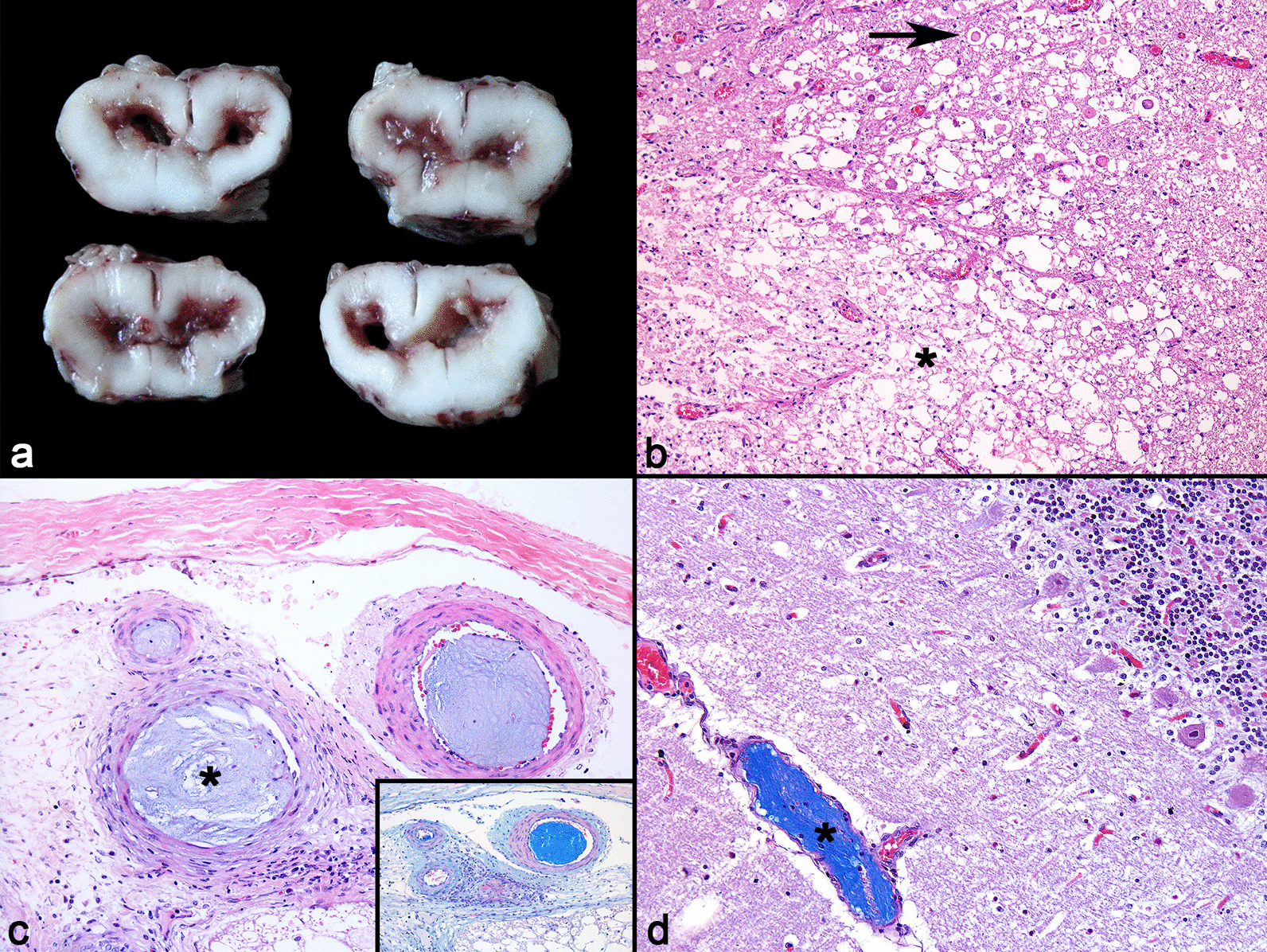
Fibrocartilaginous embolism in pigs. **a** Section of the lumbosacral spinal cord with intense brown discoloration, softening, cavitation and loss of the gray matter region. **b** Spinal cord white matter with neuropil rarefaction and vacuolation (asterisk) associated with numerous gitter cells and axonal spheroids (arrow). **c** Light basophilic compact and paucicellular material (fibrocartilaginous thrombi) occluding the lumen of arterioles of the spinal cord central branch (asterisk). HE. × 100. Inset: The intraluminal material is evidenced in blue. AB. × 100. **d** Intraluminal material stained in blue within arterioles of cerebellar leptomeninges (asterisk). AB. × 100

Three cases of CNS fibrocartilaginous embolism (FCE) were diagnosed. The first pig (pig 1) was found in lateral decubitus, with tetraparesis, and had severe lesions in the cervical segment of the spinal cord. The second pig (pig 2) with FCE was found dead but had significant skin abrasions, indicating recumbency prior to death. This pig presented uroperitoneum due to urinary bladder rupture, in addition to a focally extensive area of malacia in the lumbosacral region of the spinal cord (Fig. 6a). In the third case (pig 3), emboli were found in blood vessels of the brain and cerebellum. This pig had a previous history of fighting with other pigs the day before, and the animal developed persistent lateral decubitus, pedaling movements, convulsive episodes, and opisthotonus. All pigs diagnosed with this condition showed clinical signs of superacute or acute onset. Histopathology of the CNS revealed focal or multifocal areas of necrosis in segments of the spinal cord, especially affecting the gray matter (pigs 1 and 2) and in the cerebellum (pig 3). Wallerian degeneration was observed in the white matter (pigs 1 and 2) (Fig. 6b). In all cases, light basophilic solid material was observed within the lumen of arterioles adjacent to affected areas, especially in the meninges. This material was paucicellular and resembled the nucleus pulposus of the intervertebral discs (Fig. 6c). This material was evidenced in Alcian Blue (AB) stain (Fig. 6c insert and 6d).

### Epiphysiolysis

**Fig. 7:**
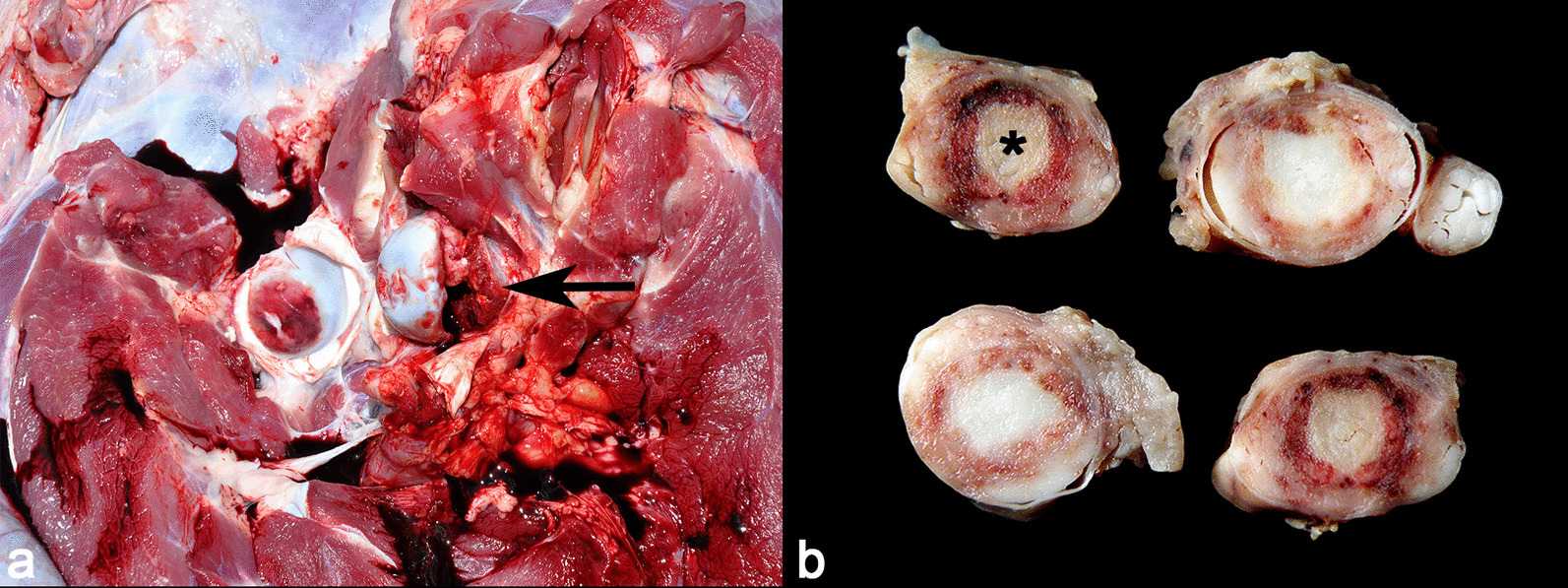
Epiphysiolysis and ascending bacterial myelitis/meningitis in pigs. **a** Fracture and separation of the femoral head (arrow), accompanied by thickening of the adjacent joint capsule by fibrous connective tissue and acetabular fossa bleeding (epiphysiolysis). **b** Cut surface of the lumbosacral segment. The spinal cord is partially effaced by a focally extensive central area containing marked accumulation of purulent material (asterisk), surrounded by a red rim (hemorrhage) (ascending bacterial myelitis)

The diagnosis of epiphysiolysis (fracture of the proximal epiphysis of the femur) was made in three pigs. Fractures occurred in the femoral head bilaterally (2/3) and unilaterally (1/3) and the pigs had severe lameness and persistent recumbency. The clinical course was subacute. Macroscopically, fracture and separation of the femoral head were observed, accompanied by thickening of the adjacent joint capsule by a white and firm tissue (fibrosis) (Fig. 7a). Microscopically, a linear fracture separating the epiphyseal plate from the metaphysis was observed, accompanied by infiltration of neutrophils and macrophages.

### Ascending bacterial myelitis/meningitis

Ascending bacterial myelitis/meningitis affected three pigs. The animals in this condition presented disease of subacute to chronic clinical course, always associated with tail-biting lesions and clinical signs of paresis/paralysis of the pelvic limbs. Macroscopically, the lesions in the tail were characterized by loss of the distal portion of tail, with crust formation and purulent content on the cut surface (Fig. 7b). The intradural space was filled with purulent material, which covered the leptomeninges and effaced partially the spinal cord parenchyma on a cut surface of the lumbosacral segment. Spinal cord histology revealed focally extensive areas of marked liquefactive necrosis affecting white and gray matter with suppurative myelitis, numerous aggregates of bacteria, and proliferation of fibrous connective tissue in surrounding areas. From these cases, two swabs from the spinal cords were sent to bacteriological culture, but there was no bacterial growth.

### Others

Diagnoses with two or fewer cases were grouped into the category "others". This group was comprised by one case of brain abscess, one case of bilateral symmetrical polioencephalitis of undetermined origin, and one case of eosinophilic meningoencephalitis compatible with water deprivation. No bacterial growth was observed in brain samples of these cases.

## Discussion

The term "downer pig" derives from "downer or downed sow syndrome". Non-ambulatory pigs, colloquially known as “downed pigs” or “downers”, occur as the clinical manifestation of numerous musculoskeletal and CNS conditions [2]. Major economic losses have been attributed to the occurrence of non-ambulatory pigs in the swine industry, with estimated losses of $54.91 per affected pig [5]. Additionally, non-ambulatory pigs represent an important issue regarding welfare standards in modern pig farms [5]. A significant number of pigs from our original study were categorized in the subset of non-ambulatory pigs, indicating the importance of this clinical manifestation as a cause of death/reason for euthanasia in the assessed pig farms.

In this study, fourteen different pathological entities were identified as the underlying disease of non-ambulatory pigs, highlighting the heterogeneity of possible etiologies for this clinical manifestation. Although numerous conditions were identified, only a few pathological entities represented the majority of cases, including chronic bacterial infections possibly occurring secondarily to tail biting lesions, systemic viral infections and primary CNS bacterial infections. The remaining conditions occurred less frequently, and were considered sporadic.

Although non-ambulatory pigs are a common problem in pig farms, thorough necropsy-based studies assessing the underlying causes for naturally occurring cases are exceedingly scarce, specially for grower-finishers. Therefore, our results add to the current knowledge on this topic and represent a valuable resource for study. Additionally, the findings of this study support the appropriateness of a necropsy-based approach with targeted ancillary testing to investigate the underlying causes for impaired ambulation in pigs.

The majority of non-ambulatory pigs were submitted for necropsy at the end of the finishing phase (150–175 days), with a predominance of chronic diseases. This age-related predisposition may occur as a result of several factors, including the prolonged time necessary for a lesion to spread from a primary site (tail lesion, for example) to distant sites, culminating in systemic lesions and clinical signs [4]; the predisposition of heavier pigs to become recumbent and succumb due to locomotor lesions [7]; and the delay of farm employees to decide for euthanasia in cases with an unfavorable prognosis.

Tail biting lesions were common and significantly associated with cases of suppurative arthritis, suppurative spondylitis, and ascendant bacterial myelitis/meningitis in this study. Tail lesions occur with greater frequency in older pigs, and are linked with stressors, including overcrowding and competition for food [9], which contributes to the later onset of the diagnosed tail biting-related diseases. In addition to these conditions, tail biting lesions are linked to increased rates of carcass condemnation at the slaughterhouse due to secondary embolic bacterial lesions [4, 10]. Tail biting lesions are also an important indicator of animal welfare in pig farms [5]. Sex predisposition for the occurrence of tail biting lesions has been previously reported for barrows [11], however, no sex predisposition was inferred in this study.

Arthritis was the most common underlying cause for non-ambulatory pigs in this study. Stifle joints are usually the most commonly affected in cases of suppurative arthritis in older pigs [12, 13], which corroborates to findings of this work. *T. pyogenes, Staphylococcus* spp. and *Streptococcus* spp. have been reported as the most important bacteria involved in suppurative arthritis in pigs [14, 15]. Interestingly, *Pasteurella multocida* was isolated in four of our cases, which is considered less common. However, studies have pointed out the septicemic involvement of *P. multocida* in cases of fibrinosuppurative arthritis [16].

Pigs with suppurative spondylitis, ascending bacterial myelitis/meningitis, FCE and vertebral fracture had clinical signs consistent with a secondary spinal cord injury, manifested by compressive or direct lesions on lower motor neurons, which can cause bladder distension and paresis/paralysis of the pelvic limbs [17–19]. In this work, cases of urinary bladder rupture associated with ascending bacterial myelitis and FCE were observed (one case each). Bacterial culture from cases of suppurative spondylitis and ascending bacterial myelitis/meningitis failed to identify a microorganism in most cases in this study, which may have occurred due to the chronicity of the lesions and frequent treatment attempts using systemic antibiotic therapy [14].

Bone fractures were occasionally seen in this study and cases were likely sporadic, as no epidemiological or morphological evidence of any predisposing factor was found, such as metabolic bone diseases or history of electrical injury. Fights and interactions among pigs may predispose older and heavy pigs to bone fractures [3]. *Streptococcus suis* and *Glaesserella parassuis* are the bacteria most commonly associated with suppurative meningitis in nursing piglets and weaners [20, 21]. In this work, suppurative meningitis was observed in younger, recently housed pigs, which may be related to stressor factors associated with adaptation to a different environment and feeding [22].

PCV-2 associated disease is multifaceted and represent one of the most important conditions in the swine species. In this study, the disease frequently led pigs to recumbency, due to muscular and CNS injuries, which often occurred concomitantly. CNS lesions in cases of PCV-2 associated diseases are classically described in pigs with PDNS and PMWS [23]. Macroscopically, the typical lesion is characterized by multiple petechiae in the cerebellum, however the entire CNS may be affected including the spinal cord [24–26]. Alternatively, muscle changes are not commonly found [27]. Histologically, muscle lesions detected in our cases were similar to those previously reported [28]. In this study, in addition to muscle necrosis, intense fibrinoid vascular degeneration was also observed. The lesions found in these cases may indicate a more acute clinical course when compared to previous descriptions [28], due to the large amount of neutrophilic infiltrate and vascular lesions. The absence of immunoreactivity in affected tissues may suggest that these changes could have occurred as a result of other mechanisms, including type III hypersensitivity reaction [29], without direct involvement of the virus in all systemic lesions [25, 28]

Viral diseases affecting the CNS are not common in the swine diagnostic routine, especially in growing-finishing pigs [30]. Possible causes of non-suppurative encephalomyelitis in pigs include Porcine Teschovirus type A (PTV) [31], Porcine Sapelovirus type A (PSV) [32], Porcine Astrovirus type 3 (PoAstV-3) [33], Suid herpesvirus 1 (SHV-1) [34], Porcine Circovirus type 2 (PCV-2) [35], Porcine Hemagglutinating Encephalitis virus (PHEV) [36], and Porcine Reproductive and Respiratory Syndrome virus (PRRSV) [37]. The findings observed in our cases are highly suggestive of infection by a neurotropic virus, such as PSV, PTV and PoAstV-3 [32, 33, 38]. Some of these viruses have been identified in fecal samples in Brazilian pig farms [39, 40], and more recently as a cause of clinical disease in a herd in southern Brazil [38]. In this study, no complementary tests were performed to determine the etiology involved in cases of non-suppurative encephalomyelitis.

Ischemic myelopathy due to FCE is a disease clinically characterized by acute, non-painful, and non-progressive neurological dysfunction. Sporadic reports of FCE in pigs have been documented leading to ischemic infarction in the spinal cord or brain [41–44]. These present findings suggest that FCE may be more common than previously believed in pigs. The pathogenesis of FCE in animals is unclear, nevertheless, some factors may be related to the occurrence of this condition, such as degeneration of the dorsal annulus of the intervertebral disc, herniation of the disc or extrusion of the nucleus pulposus, in addition to minor traumas in the region [45], persistence of embryonic remnant vessels within the nucleus pulposus [46], and discospondylitis [41]. At necropsy, no evidence of preceding injuries affecting the vertebral column and intervertebral discs were found in our cases. It is believed that the habit of fighting in pigs, as well as hyperstimulation of pigs during handling or transportation may be associated with small vascular traumas, predisposing pigs to FCE. In one of the cases of this study, FCE affected the cerebellum. This lesion distribution is not commonly observed in cases of FCE, with rare reports in veterinary literature [47]. In this case, it is suggested that the nucleus pulposus may have originated from the cervical segment of the vertebral column, and the material entered small arteries, and occluded the lumen of vertebral arteries retrogradely, similarly to previous descriptions [42, 48].

Epiphysiolysis affected pigs at the end of finishing phase. This disease usually occurs in gilts between four and eight months of age and consists of aseptic fracture of the femoral neck [49]. The cause has been associated with excessive tension in the hip joint due to excessive weight, which leads to fracture of the physeal region. Clinical lameness is usually severe and of sudden onset, which may be unilateral or bilateral as observed in the pigs of this study [50].

## Conclusions

Numerous conditions leading to the clinical manifestation of non-ambulatory pigs were identified in growing-finishing farms in this study. Clinical cases occurred predominantly at the end of the finishing phase. Diagnosed entities included pathologies of the locomotor system (suppurative arthritis, epiphysiolysis, and fractures), CNS (suppurative meningoencephalitis and non-suppurative encephalomyelitis, ascending bacterial myelitis, fibrocartilaginous embolism, brain abscess, polioencephalitis of non-determined origin, and eosinophilic meningoencephalitis), and also multisystemic pathologies such as PCV-2 associated diseases.

Tail biting lesions were an important predisposing factor and acted as the probable primary site of infection for cases of suppurative arthritis, spondylitis, and ascending bacterial myelitis. Neurologic and muscular lesions were common in cases of PCV-2 associated diseases, reinforcing the importance of this conditions as differential diagnosis in cases of non-ambulatory pigs. The necropsy examination is an essential tool to monitor the underlying causes of non-ambulatory pigs, generating essential information on infectious and non-infectious diseases affecting commercial herds. Information obtained from similar investigations is paramount to improve field diagnostic efforts, mitigate economic losses and improve animal welfare in the swine industry.

## Material and methods

A larger study conducted by our research group has previously assessed the causes of mortality in grower-finishers in two pig farms in southern Brazil [8]. In this necropsy-based study, we evaluated 610 pigs, 76 (12.5%) of which were non-ambulatory pigs. Now, we further explore the postmortem examination findings of this subset of cases.

Post-mortem examinations and sampling were carried out in two commercial pig farms (Farm A and B) located in the western region of the state of Santa Catarina, Brazil. Farm A and B were visited four times, each visit lasted 12 days, with eight visits in total. Both farms housed 70-day-old weaners, and pigs were transferred to sow farms or sent to slaughter by the end of the finishing phase (175 days of age). During the visits, necropsy procedures were carried out in all pigs that died spontaneously or were euthanized by farm employees. A total of 610 pig necropsies were performed, and results have been published elsewhere [8].

In this study, we focus on the subset of cases of non-ambulatory pigs. All pigs included in this study had a history of impaired ambulation, including difficulty to stand up and walk and prolonged sternal or lateral recumbency, which was the primary cause of death or reason for euthanasia. Information provided by farm employees consisted of age, sex, and clinical history, including disease evolution, administered medications, and response to treatment in affected pigs. Body Condition Score (BCS) was inferred through visual evaluation. BCS was graded from 1 to 5; BCS 1 comprised cachectic pigs and BCS 5 comprised pigs with significant fat coverage [51]. The clinical evolution in each case was classified as peracute (0–24 h), acute (24–96 h), subacute (4–14 days), and chronic (> 14 days) [52].

Postmortem examinations were carried out, gross lesions were recorded, and organ fragments were systematically collected and fixed in 10% formalin solution. Subsequently, tissues were routinely processed for the preparation of histological slides, which were stained by hematoxylin and eosin (HE), in addition to Alcian blue stain in selected cases. When there was a history of decubitus, locomotor deficits, and/or neurological clinical signs, the necropsy procedure included an evaluation focused on the CNS and musculoskeletal systems. This evaluation comprised a detailed inspection of the appendicular and axial skeletons, the skeletal muscles of fore and hindlimbs, the appendicular synovial joints, the brain, and spinal cord, with subsequent collection of samples of these tissues for histology. When infectious agents were suspected to be involved in the lesions, fragments of organs and body fluids were collected, kept refrigerated, and sent within 24 h for bacteriological, biochemical, and molecular examinations.

Samples sent for bacterial culture were inoculated in blood agar plates (BA) and MacConkey agar (MC) and incubated at 36 °C ± 1 °C for 18–48 h in an aerobic atmosphere [8]. Biochemical characterization of the colonies for the identification of bacteria was performed as previously described [53]. For the detection of Porcine Circovirus type 2 (PCV-2), pool samples of lymph nodes of six cases of PCV-2 associated diseases were submitted to PCR according to the methodology previously described [54]. Additionally, immunohistochemistry (IHC) was performed to detect PCV-2 antigens in selected sections of lymph nodes, skeletal muscle, and CNS in suspect cases, according to the methodology previously described [55].

The proportion of pigs diagnosed with a tail-biting related disease was compared between two categories: pigs with tail-biting lesions (yes) and pigs with no tail-biting lesions (no). Tail-biting related diseases encompassed cases of suppurative arthritis, suppurative spondylitis, and ascending bacterial myelitis/meningitis. The proportion of pigs with tail-biting lesions was also compared between sex categories (barrows or females). The comparison used Pearson's Chi-square test, except if the variables showed an expected number of observations less than 5 in 25% of the cells of the contingency table, then Fisher's Exact test was used instead.

The odds ratio (OR) and confidence interval (95% CI) of tail-biting-related diseases were calculated from the odds that a case (with tail-biting lesions) was exposed to the odds that a control (without tail-biting lesion) was exposed using logistic regression. The significance level chosen was 5% for all hypothesis tests. Statistical analysis was performed using commercial software.

## Data Availability

Besides the presented data, raw data can be shared upon reasonable request by contacting the corresponding author and will require prior acceptance from herd owners.

## Abbreviations

AB: Alcian Blue stain
BA: Blood agar plates
BCS: Body Condition Score
CNS: Central Nervous System
FCE: Fibrocartilaginous Embolism
PHEV: Porcine Hemagglutinating Encephalomyelitis Virus
IHC: Immunohistochemistry
MC: MacConkey agar
NEM: Non-suppurative encephalomyelitis
OR: Odds ratio
PoAstV-3: Porcine Astrovirus type 3
PCV-2: Porcine Circovirus type 2
PDNS: Porcine Dermatitis and Nephropathy Syndrome
PRRSV: Porcine Reproductive and Respiratory Syndrome virus
PSV: Porcine Sapelovirus type A
PTV: Porcine Teschovirus type A
PMWS: Post-weaning Multisystemic Wasting Syndrome
SHV-1: Suid herpesvirus 1
